# Effects of lactic acid bacteria and molasses on fermentation dynamics, structural and nonstructural carbohydrate composition and *in vitro* ruminal fermentation of rice straw silage

**DOI:** 10.5713/ajas.18.0543

**Published:** 2018-11-27

**Authors:** Jie Zhao, Zhihao Dong, Junfeng Li, Lei Chen, Yunfeng Bai, Yushan Jia, Tao Shao

**Affiliations:** 1 Institute of Ensiling and Processing of Grass, College of Agro-grassland Science, Nanjing Agricultural University, Nanjing 210095, China; 2 Jiangsu Academy of Agricultural Sciences, Nanjing 210014, China; 3 Key Laboratory of Forage Cultivation, Processing and High Efficient Utilization of Ministry of Agriculture, Inner Mongolia Agricultural University, Hohhot 010018, China

**Keywords:** Rice Straw, Molasses, *Lactobacillus plantarum*, Silage, *In vitro* Fermentation

## Abstract

**Objective:**

This study was to evaluate the fermentation dynamics, structural and nonstructural carbohydrate composition and *in vitro* gas production of rice straw ensiled with lactic acid bacteria and molasses.

**Methods:**

Fresh rice straw was ensiled in 1-L laboratory silos with no additive control (C), *Lactobacillus plantarum* (L), molasses (M) and molasses+*Lactobacillus plantarum* (ML) for 6, 15, 30, and 60 days. After storage, the silages were subjected to microbial and chemical analyses as well as the further *in vitro* fermentation trial.

**Results:**

All additives increased lactic acid concentration, and reduced pH, dry matter (DM) loss and structural carbohydrate content relative to the control (p<0.05). The highest organic acid and residual sugar contents and lignocellulose reduction were observed in ML silage. L silage had the highest V-score with 88.10 followed by ML silage. L and ML silage improved *in vitro* DM digestibility as compared with other treatments, while *in vitro* neutral detergent fibre degradability (IVNDFD) was increased in M and ML silage (p<0.05). M silage significantly (p<0.05) increased propionic acid (PA) content and decreased butyric acid content and acetic acid/PA as well as 72-h cumulative gas production.

**Conclusion:**

The application of ML was effective for improving both the fermentation quality and *in vitro* digestibility of rice straw silage. Inclusion with molasses to rice straw could reduce *in vitro* ruminal gas production.

## INTRODUCTION

Rice (*Oryza sativa*, L.) harvesting produces the largest amount of crop residues worldwide each year. Approximately 21 Mt/yr of rice straw was produced accounting for 47% of the total crop residue in China [1]. However, a high proportion of rice straw has been left unused or burnt directly, wasting resource and causing environmental pollution, indicating an urgent need for proper disposal of rice straw. Meanwhile, because of the seasonality of straw harvesting and annual supply of feedstuffs needed, long-term effective storage of harvested straw is required. Haymaking of rice straw is impractical because of the low feed value resulting from its structural characteristics and long drying process. Ensiling as a promising technology is applicable for straw conservation in a humid climate and has been used to treat straw waste and supply year-round availability of feeds.

Rice straw is difficult to ensile due to its hollow stem, low WSC and less epiphytic lactic acid bacteria (LAB) [2]. Thus, exogenous LAB and fermentable substrate are commonly applied to improve the feeding value of such low-quality roughages. *Lactobacillus plantarum* (*L. plantarum*) as the dominant type of silage additive has been commonly applied worldwide for decades. Adequate *L. plantarum* would ensure extensive fermentation and efficient utilization of substrates in ensiled materials. It was reported that that the addition of *L. plantarum* improved the quality of sorghum straw silage [3]. Molasses is a by-product of sugar industries and rich in soluble carbohydrate contents, especially for sucrose and glucose, which provides a low-cost sugar source for LAB and compensates for the sugar deficiency of rice straw. Chen et al [4] reported that applying molasses not only stimulated lactic acid fermentation but also promoted degradation of structural carbohydrate as compared with other treatments.

Conventional forages, such as alfalfa, are commonly used as silage material and little effort has been devoted to developing the rice straw silage. Consequently, information, availability and application of these silage additives on this material are still limited. The aim of this study was to assess the effects of molasses and/or *L. plantarum* on fermentation quality, structural and nonstructural carbohydrate composition and *in vitro* digestibility of fresh rice straw silage.

## MATERIALS AND METHODS

### Silage additives

*Lactobacillus plantarum* (L, Ecosyl MTD/L, Ecosyl Products Ltd., Stokesley, North Yorkshire, UK) was inoculated and cultured in MRS broth medium according to the recommendations of the manufacturer. Molasses was a by-product of sugar industry and obtained from JiaFurui Biological Technology Co., Ltd. (Nanjing, Jiangsu, China).

The *L. plantarum* inoculant was applied at a level of 10^6^ colony forming units (cfu) per gram of fresh weight (FW), and molasses was applied at 4% FW.

### Silage preparation

Fresh rice straw was collected from Nanjing Branch of Chinese National Centre for Rice Improvement in Jiangsu Academy of Agricultural Science (32.04°N, 118.88°E, 20 m asl, Jiangsu, China), leaving the stubble of 10 cm.

The straw was chopped into lengths of 2 to 3 cm with a fodder chopper followed by manual mixing and ensiling with: i) no additive (C), ii) *L. plantarum* (L), iii) molasses (M), and iv) molasses+*L. plantarum* (ML). Additives were diluted with deionised water to an equivalent of 20 mL/kg FW and spray mixed into the freshly chopped samples. Same amount of deionised water was applied to the control. Thereafter, approximately 550 g treated material was tightly packed into 1-L laboratory silos (polyethylene bottle with diameter of 9.5 cm and height of 18.7 cm, Lantian biological experimental instrument Co., Ltd, Jiangsu, China) and stored at the ambient temperature (22°C to 28°C) after being sealed with screw tops and plastic tape. Quintuplicate per treatment were opened on 6, 15, 30, and 60 days after ensiling, respectively.

### Chemical and microbial analyses

#### Analysis of raw material

The fresh material was immediately sampled for the determination of crude protein (CP), crude ash, buffering capacity (BC), and the counts of epiphytic microorganisms. Total nitrogen (TN) was determined by Kjeldahl nitrogen analyser (Kjeltec 8200; FOSS, Höganäs, Sweden), and the CP was calculated as TN×6.25. Ash was measured by incinerating in a muffle furnace at 550°C for 4 h. The BC was determined according to the method described by Playne and Mcdonald [5]. The plate counting method and the cfu were used for the enumeration of epiphytic microorganism populations. The samples (10 g) homogenized with 90 mL sterilized saline solution (8.50 g/L NaCl) was serially diluted from 10^−1^ to 10^−6^. The LAB, aerobic bacteria, moulds and yeasts were counted on MRS agar medium, nutrient agar medium and potato dextrose agar medium at 30°C for 2 to 3 days, respectively. The ensilability of rice straw was assessed by calculating the fermentation coefficient (FC) according to formula described by Yitbarek and Tamir [6].

#### Analysis of liquid samples

Fresh or ensiled rice straw was divided into two subsamples. The first subsample was blended with distilled water at ratio of 1:3 and stored at 4°C macerating for 24 h to obtain cold extract. Then, the cold extract was filtered through two layers of cheesecloth and a Whatman filter paper (pore size of 11 μm, Xinhua Co., Hangzhou, China). The filtrate was stored at −20°C for subsequent determination of pH, ammonia nitrogen (NH_3_-N), and organic acid concentrations. The pH was measured with a glass electrode pH meter (HANNA pH 211; Hanna Instruments Italia Srl, Villafrance Padovana, Italy). The NH3-N was determined by the phenol-hypochlorite reaction method of Broderick and Kang [7]. The organic acids and ethanol analyses were conducted in high performance liquid chromatography system (1260 HPLC, Agilent Technologies, Inc., Waldbronn, Germany) equipped with a refractive index detector (column: Carbomix H-NP5, Sepax Technologies, Inc., Newark, DE, USA; eluent: 2.5 mM H_2_SO_4_, 0.5 mL/min; at temperature 55°C with 30 min run time).

The V-score method [8] was adopted to evaluate the silage quality using a 100-point scale as below: <60 (bad), 60 to 80 (moderate) and 81 to 100 (good). V-score = Y_N_+Y_A_+Y_B_, where Y_N_ is calculated from the NH_3_-N content (% TN), Y_A_ is calculated from the acetate+propionate contents (% dry matter [DM]), and Y_B_ is calculated from the butyrate content (% DM).

#### Analysis of solid samples

The second subsample was freeze dried by a vacuum freeze dryer to determine DM content. The solid samples were ground to pass 1-mm screen with laboratory knife mills (FW100, Taisite Instrument Co., Ltd., Tianjin, China) and stored for later analysis of neutral detergent fibre (NDF), acid detergent fibre (ADF), acid detergent lignin (ADL), water soluble carbohydrates (WSC) and monosaccharide compositions. Further analysis of CP and ash contents was carried out in 60-day silage samples. The WSC was determined via a modified phenol-sulfuric acid method [9]. The contents of NDF, ADF, and ADL were measured using the procedures of ANKOM filter bag technique by ANKOM 200i fibre analyser (ANKOM Technologies, Inc., Fairport, NY, USA). The mono- and disaccharide compositions (glucose, xylose, fructose, and sucrose) were determined according to the method of Desta et al [10].

### *In vitro* incubation of 60-day silages

*In vitro* fermentation was conducted in serum bottles following the Contreras-Govea et al [11] method with some modifications. The inoculum (rumen fluid) was derived from various locations within the rumen of 2 Holstein cows before morning feeding. The cows were fed the diet based on corn silage at 1.2 times of the maintenance level [12]. Rumen fluid was filtered, moved to laboratory, and stored at 39°C in a water bath. Prior to use, the rumen fluid was mixed with buffer solution at ratio of 1:2 (v/v) as described by Menke [13]. The whole operation process was carried out under continuous flushing with CO_2_.

Ground samples (1 g) were placed in filter bags (F57; ANKOM Technology, Macedon, NY, USA) that were washed with acetone, dried at 55°C for 24 h and weighted previously. Then each bag was heat-sealed and put into each preheated serum bottle (120 mL capacity) with 60 mL inoculum under CO_2_ at 39°C. Triplicates per treatment were performed in two separate *in vitro* experimental runs and the blank was 3 serum bottles with only inoculum added. The gas volume was determined at 4, 8, 12, 24, 48, and 72 h of incubation by pressure transducer technique according the method of Jiao [14] and corrected with blank bottles. After incubation, the inoculum in the bottle was filtered through a pre-dried and weighted nylon bag (200 mesh). The filtrate was collected for analyses of ruminal pH and volatile fatty acids with the method as described above. The residual samples were gently rinsed with cold tap water and dried at 65°C for 48 h to determine *in vitro* digestibility of dry matter (IVDMD), neutral detergent fibre (IVNDFD), and acid detergent fibre (IVADFD). IVDMD, IVNDFD, and IVADFD were calculated based on the differences in their respective weight before and after incubation.

### Statistical analysis

The data was subjected to two-way analysis of variance with the fixed effects of additives, ensilage period and additives× ensilage period using the general linear model procedure of SAS rev. 9.1. Microbial data were normalised by the log10-transformation on a FW basis. Tukey’s multiple comparison was used to determine the statistical difference between means, and the level of significance was set at p<0.05.

## RESULTS

### Chemical and microbial composition of fresh material

The chemical and microbial composition of silage materials are shown in Table 1. The fresh rice straw had high DM, structural carbohydrate content and FC, and low CP content and BC. The initial pH value was 6.43. The epiphytic LAB on rice straw were less than 1.0×10^5^ cfu/g FW. The number of aerobic bacteria were higher than that for moulds and yeasts, which were more than 1.0×10^6^ cfu/g FW.

### Fermentation quality of rice straw silages

Table 2 illustrates the dynamics of organic acids and ethanol contents of rice straw silage during the ensiling. Treatments, ensiling days and their interaction significantly affected these parameters (p<0.05). All additives significantly (p<0.05) increased lactic acid (LA) concentration and LA/acetic acid (AA), while decreased (p<0.05) AA, butyric acid (BA) and ethanol concentration of the silage. As compared with other treatments, *L. plantarum* addition further increased the LA concentration and LA/AA, decreased the amounts of AA and ethanol. The LA concentration in all silages soared to reach a peak at day 30 then followed by a sharp drop at end of ensiling (p< 0.05). The highest LA concentration was recorded in ML silage with the value of 103.63 g/kg DM. No or negligible amounts of BA was observed in all silages, except for the control (2.51 g/kg DM).

**Figure 1:**
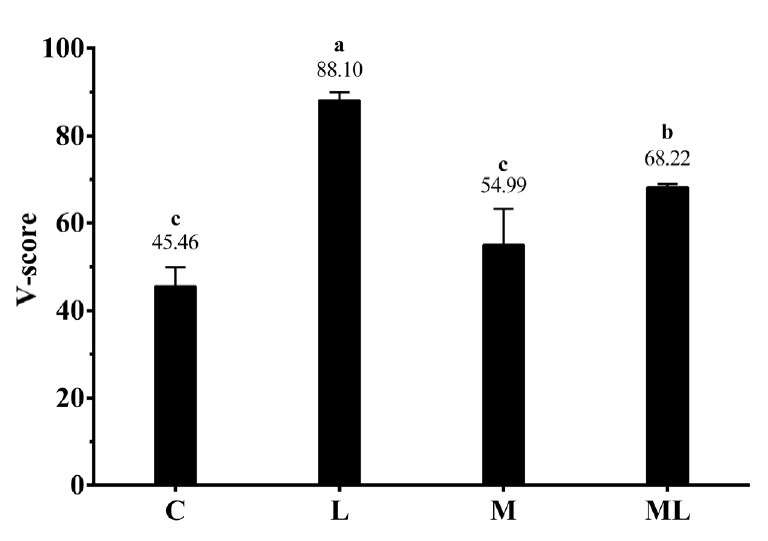
V-score of 60-day rice straw silages. DM, dry matter. Treatments: C, no additive control; L, *Lactobacillus plantarum*; M, molasses; ML, molasses+*Lactobacillus plantarum* (n = 5, bars indicate standard error of the means). Means with different small letters show significant difference among treatments at p<0.05.

The pH, DM, DM loss, and NH_3_-N of rice straw silages are listed in Table 3. The DM and DM loss were significantly affected by additives, ensiling days and their interaction (p<0.05). All additives, especially L treatment, significantly (p<0.05) decreased pH, DM loss, and NH_3_-N contents of rice straw silages. The pH values of the additive silages rapidly decreased during the first 15 days of ensiling then tended to increase. Meanwhile, L and ML silages always maintained a lower pH value below 4.15 during ensiling. The DM was significantly decreased (p< 0.05) along the ensiling process accompanied by constantly increase of DM loss. Molasses treated silages showed relative high (p>0.05) DM contents at day 6 of ensiling, while inoculated silage preserved higher (p<0.05) DM contents at the end of ensiling. The NH_3_-N content exhibited a continuously uptrend during the whole ensiling. All additives, except for M treatment, significantly increased (p<0.05) V-score of rice straw silage (Figure 1).

### Structural carbohydrate compositions of rice straw silage

Structural carbohydrates compositions of rice straw silage are given in Table 4. The effects of treatments and ensiling days were significant (p<0.05) on NDF, cellulose and hemicellulose components of rice straw silages. All measured structural carbohydrate fractions, except for ADL, were reduced (p<0.05) in additive silages and showed a continuous decrease throughout the ensiling. *L. plantarum* and its combination with molasses further decreased the structural carbohydrate contents relative to sole addition, and minimum content of structural carbohydrate was observed in ML silage (p>0.05). After 30 days of ensiling, ML silage showed lower (p<0.05) contents of NDF and hemicellulose than other silages during ensiling. In addition, the larger decline of NDF than that of ADF occurred in all silages during ensiling.

### Nonstructural carbohydrate compositions of rice straw silage

**Figure 2:**
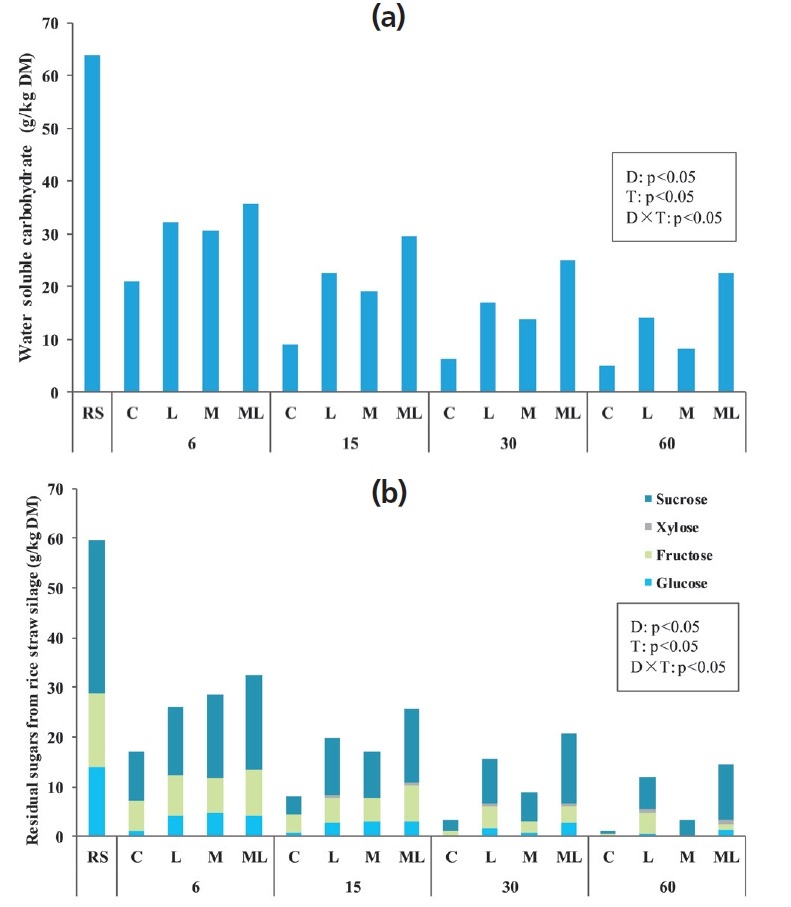
(a) Water soluble carbohydrates and (b) residual sugars (glucose, fructose, xylose and sucrose) of fresh and ensiled rice straw. DM, dry matter. Treatments: RS, fresh rice straw; C, no additive control; L, *Lactobacillus plantarum*; M, molasses; ML, molasses+*Lactobacillus plantarum* (n = 5). The effects of ensilage days (D), treatments (T) and their interaction (D×T) were significant at p<0.05.

As shown in Figure 2, treatments, ensiling days and their interaction significantly affected the nonstructural carbohydrate contents of rice straw silage (p<0.05). The additive-treated silages preserved significantly higher (p<0.05) concentrations of nonstructural carbohydrate than the control silage. All nonstructural carbohydrates tended to decrease along the storage period (p<0.05), except for xylose with an upgrade tendency. The WSC concentrations were significantly higher (p<0.05) in L and ML silage than other treatments after 6 days of ensiling (Figure 2a). Interestingly, the type of additives caused significant differences (p<0.05) in residual sugars contents. High sucrose concentration was determined in M and ML silages during the first 6 days of ensiling, and trace amounts of xylose were observed in L and ML silage after 15 days of ensiling.

### Crude protein, ash and *in vitro* ruminal fermentation parameters of 60-day rice straw silage

The CP and ash contents of rice straw silage after 60 days of ensiling are presented in Table 5. The additive-treated silages had significantly higher (p<0.05) CP content than the C silage, with the highest value in ML followed by M then L. The ash content of L silage was lower (p<0.05) than that of other treatments and there was no significant difference among C, M, and ML silages. ML addition significantly (p<0.05) improved IVDMD and IVNDFD of resulting silage, while no notable differences were found in IVADFD among the four silages.

**Figure 3:**
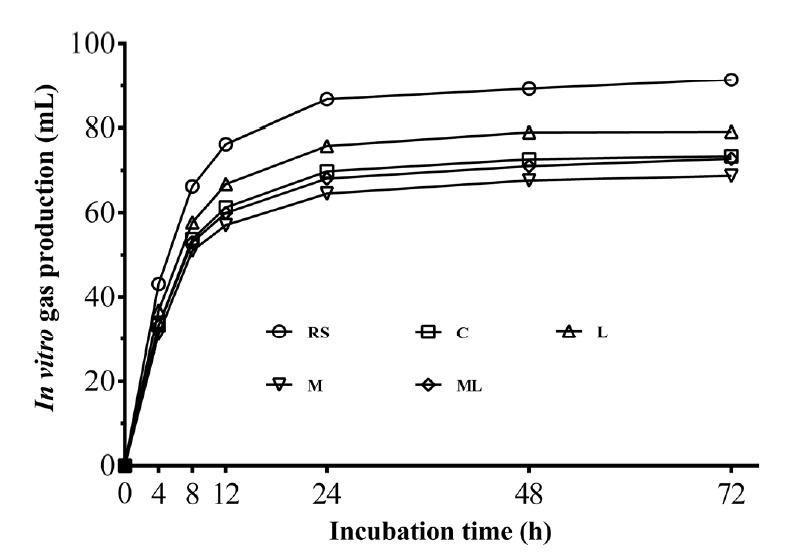
Gas production profiles (mL gas/g DM) from *in vitro* fermentation of rice straw silages for 72 h. DM, dry matter. Treatments: RS, fresh rice straw; C, no additive control; L, *Lactobacillus plantarum*; M, molasses; ML, molasses+ *Lactobacillus plantarum* (n = 5).

Table 6 presents ruminal fermentation characteristics of 60-day rice straw silages after 72 h incubation. The additives effect was significant on ruminal pH, AA, propionic acid (PA) and BA (p<0.05). Molasses addition (M and ML) significantly increased PA content and decreased BA content and AA/PA (p<0.05). As shown in Figure 3, all silages had less gas production as compared with fresh rice straw (p<0.05). L silage had higher 72-h cumulative gas production (GP_72_) than the other treatments (p<0.05). While the gas production of M (p<0.05) and ML (p>0.05) was lower than that of the control.

## DISCUSSION

### Analysis of raw materials

The rice straw used in this study contained high FC (>35), low BC, relatively proper DM and WSC content, which theoretically are suitable for natural fermentation [15]. However, the epiphytic LAB on rice straw is too low (<1.0×10^5^ cfu/g FW) to dominate fermentation [16]. Furthermore, the rice straw had high contents of structural carbohydrate, with NDF and ADF accounting for approximately 71% and 41% of DM, respectively. Consequently, rice straw presents difficulties for long-term preservation through natural fermentation.

### Analysis of fermentation quality of rice straw silages

All inoculated silages were well fermented, with low pH and NH_3_-N content, high LA contents as well as V-scores. This indicated that *L. plantarum* was more effective than molasses to improve the fermentation quality of rice straw silages. Actually, most epiphytic LABs were cocci, which produce LA during the initial stages of ensiling [16]. Mcdonald et al [15] reported that about 80% were found as leuconostocs, and lactobacilli were the least common on silage materials. Wang et al [17] reported that some lactobacilli (such as *Lactobacillus helveticus*) cannot live well on the surface of rice straw. These findings may explain the low LA contents in M and C silage. If lactobacilli cannot dominate the fermentation, undesired microorganisms will prevail, which is consistent with the BA and high NH_3_-N observed in this study at the end of ensiling.

*L. plantarum* can ferment a wide source of substrates and quickly produce large amounts of LA. Indeed, *L. plantarum* addition significantly increased LA concentrations with a concomitant decrease in pH. The sharp decline of LA after 30 days could be explained by LA being converted to AA by heterofermentative lactic bacteria at the later stage of fermentation [15]. Heterofermentative LAB strains can degrade LA into several metabolites, mainly AA [18]. Differently, the pH values of L and M silages tended to increase after 15 days of ensiling. This result is possibly due to the alkalinisation effect of NH_3_-N. The AA concentration showed an uptrend along the ensiling period, and LA/AA declined after 6 days of ensiling, indicating an obvious shift from homofermentation to heterofermentation at early stage of ensiling [15].

High levels of ethanol were detected in all silages and this could be explained by the high DM of rice straw and activity of yeasts. Ethanol is commonly formed in high DM silage [19]. Moreover, less ethanol in L and ML silages may be related to an inhibition of yeasts resulting from the inoculation with *L. plantarum*. Driehuis et al [20] also reported that the addition of inoculant significantly decreased ethanol concentration in high DM silage as compared with the control. BA was absent or detected in negligible amounts except for the control, suggesting that low pH in additive-treated silages inhibited clostridial fermentation. Clostridial fermentation signified protein degradation, dry matter loss and energy wastage. Similarly, high NH_3_-N content and DM loss was also observed in C silage (p<0.05). The DM content showed a continuous downward trend, with considerable DM loss recorded in C silage followed by M silage. This could be attributed to the breakdown of nutrients caused by clostridial spoilage or heterofermentation [15].

### Analysis of structural carbohydrates compositions of rice straw silages

The structural carbohydrate compositions of rice straw silage are presented in Table 4. The ensiled rice straw had higher structural carbohydrate contents than untreated rice straw (Table 1), probably due to the high DM loss, which primarily evolved from non-fibre fractions. The effects of LAB inoculants on degradation of structural carbohydrate are uncertain. Muck [21] reported that LAB cannot effectively use fibre as an energy source to produce LA. However, all additives, including *L. plantarum*, reduced the structural carbohydrate contents compared with the control in this study. This could be explained that more soluble components retained in additive-treated silages indirectly reduced relative proportions of structural carbohydrates. In addition, the acid solubilization of hemicellulose evinced by organic acid (particularly LA) in inoculated silage is also considered. Dewar et al [22] concluded that after ensiling of 7 to 28 days, structural carbohydrates could be degraded by acid hydrolysis at low pH. Interestingly, the ADL content exhibited an uptrend over the course of ensiling, which could be explained by the high loss of DM. In fact, ensilage cannot affect the ADL content.

### Analysis of nonstructural carbohydrates compositions of rice straw silages

Within the initial 6 days of ensiling, the maximum consumption of WSC (Figure 2a) was accompanied by small amounts of fermentation products (Table 2) in C silage, suggesting that the readily available substrates were mainly consumed by plant respiration and aerobic bacteria in the control.

The higher residual sugar in inoculated (L and ML) silages could be attributed to an inhibition of undesired microbial growth and acid hydrolysis of available structural carbohydrates, which were reflected in the low pH and xylose production. Hemicellulose, also known as polyose, is formed by various components including a backbone of xylans and arabinose side chains [23], which can be decomposed into xylose and arabinose. Similar to the study of Shao et al [24], a larger reduction was observed in glucose than that of other sugars. This result indicated that glucose could be the more favourable fermentation substrate for LAB during ensiling.

### Analysis of crude protein, ash and *in vitro* ruminal fermentation parameters of 60-day rice straw silages

Low CP content observed in the control could be attributed to the higher residual air in coarse and hollow stems of rice straw, which facilitated proteolytic bacteria growth during ensiling. Higher CP observed in M-treated silages than fresh material could ascribe to DM loss and part of nitrogen in molasses. On the other hand, L and ML application induced rapid acidification thereby suppressing protein degradation by undesired microorganisms. The fluctuation of ash content could be related to DM loss since ash was expressed as a percentage of DM.

*In vitro* gas production is commonly used as an indicator for efficiency of rumen degradability and predicts the metabolizable energy of animal feed [11]. In this study, gas production of all silages was reduced relative to the fresh rice straw, indicating that *in vitro* ruminal gas production can be lowered by ensiling. Moreover, molasses addition further decreased GP_72_ of resulting silages. This result is difficult to explain but probably due to the molasses-treated rice straw silage altered ruminal short-chain fatty acid production. Yulistiani et al [25] reported that adding molasses increased ruminal PA proportion, and decreased acetic/PA and gas production of fermented fibrous material *in vitro* trial. Also, study of Xia et al [26] followed a similar result with higher PA content and lower gas production in molasses-treated wheat silage. This is consistent with the results of this study. The rumen gas, such as hydrogen and methane, can be reduced by the shift of ruminal fermentation pattern from acetic to propionic type [27]. Thus, further study is needed to clarify and explain this phenomenon.

Digestibility has gained wide acceptance in the evaluation of feed nutritional value and intake. Cao et al [28] reported that IVDMD was higher in inoculated silage, because LAB addition had less DM loss during silage fermentation. Similarly, L and ML silage had higher IVDMD than other treatments in this study. Previous studies [29] showed that inoculation of LAB at ensiling could improve IVNDFD of grass silage in mixtures with legume or corn silage. While a low IVNDFD was observed in L silage. This result could relate to the silage fermentation results in hydrolysis of the hemicellulose which is acid labile at strong acid condition [30], and then less NDF was available for rumen microbial degradation. The ML addition effectively improved *in vitro* digestibility of rice straw silage, indicated by higher DMD, NDFD, ADFD.

In conclusion, additives are necessary to avoid spoilage and enhance the fermentation stability of fresh rice straw silage. *L. plantarum* was more effective than molasses to improve the silage quality of rice straw. Molasses addition reduced *in vitro* gas production of rice straw silage. The application of both *L. plantarum* and molasses is recommended to enhance its fermentation quality, nutritive characteristics and *in vitro* digestibility. Rice straw can be well preserved by ensiling with additives, thereby providing a continuous roughage source for ruminant livestock in rice production area.

## Figures and Tables

**Table 1:** Chemical and microbial compositions of silage materials

Items	Rice straw	Molasses
pH	6.43	-
Dry matter (g/kg FW)	417.96	617.37
Crude protein (g/kg DM)	61.24	29.53
Water soluble carbohydrate (g/kg DM)	63.79	647.29
Neutral detergent fibre (g/kg DM)	603.04	-
Acid deterge fibre (g/kg DM)	377.10	-
Buffering capacity (mEq/kg DM)	39.71	-
Fermentation coefficient	54.90	-
Ash (g/kg DM)	118.41	-
Lactic acid bacteria (log_10_ cfu/g FW)	4.54	-
Aerobic bacteria (log_10_ cfu/g FW)	6.32	-
Moulds (log_10_ cfu/g FW)	3.97	-
Yeasts (log_10_ cfu/g FW)	4.48	-

FW, fresh weight; DM, dry matter; mEq, milligram equivalent, cfu, colony-forming units.

**Table 2:** Effect of additives and ensiling days on organic acid and ethanol composition of rice straw silages

Items	Treatment1)	Ensiling days				Means	SEM	Significance2)		
	
		6	15	30	60			D	T	D×T
Lactic acid (g/kg DM)	C	16.22^c^^B^	26.26^c^^B^	37.80^d^^A^	22.77^d^^B^	25.76^d^	3.46	*	*	*
	L	51.80^ab^^B^	64.06^ab^^AB^	81.12^b^^A^	59.97^b^^B^	64.24^b^				
	M	42.46^b^^B^	50.59^b^^AB^	60.39^c^^A^	40.39^c^^B^	48.46^c^				
	ML	55.31^a^^D^	73.65^a^^C^	103.63^a^^A^	88.68^a^^B^	80.32^a^				
Acetic acid (g/kg DM)	C	8.07^a^^D^	13.03^a^^C^	22.38^a^^B^	27.46^a^^A^	17.73^a^	0.91	*	*	*
	L	5.88^ab^^C^	7.94^b^^C^	12.25^b^^B^	15.38^c^^A^	10.36^c^				
	M	7.55^ab^^C^	9.94^b^^C^	14.47^b^^B^	20.57^b^^A^	13.13^b^				
	ML	5.44^b^^D^	7.52^b^^C^	12.49^b^^B^	17.34^c^^A^	10.70^c^				
Butyric acid (g/kg DM)	C	ND	0.36	1.68	2.51	1.14^a^	0.11	*	*	*
	L	ND	ND	ND	ND	0^b^				
	M	ND	ND	ND	0.62	0.15^b^				
	ML	ND	ND	ND	ND	0^b^				
Ethanol (g/kg DM)	C	12.28^a^^B^	10.76^a^^B^	21.41^a^^A^	28.60^a^^A^	18.26^a^	0.87	*	*	*
	L	7.15^bc^^B^	8.72^b^^B^	11.76^b^^A^	13.44^bc^^A^	10.27^c^				
	M	8.32^b^^D^	10.63^a^^C^	13.77^b^^B^	16.32^b^^A^	12.26^b^				
	ML	5.49^c^^C^	6.68^c^^BC^	7.45^c^^AB^	8.21^c^^A^	6.96^d^				
Lactic acid/acetic acid	C	2.00^c^^A^	2.04^c^^A^	1.69^d^^A^	0.83^d^^B^	1.64^d^	0.45	*	*	*
	L	8.81^a^^A^	8.35^ab^^A^	6.68^b^^AB^	3.90^b^^B^	6.93^b^				
	M	5.89^b^^A^	5.10^bc^^A^	4.20^c^^AB^	1.96^c^^B^	4.29^c^				
	ML	10.20^a^^A^	9.83^a^^AB^	8.30^a^^B^	5.12^a^^C^	8.36^a^				

DM, dry matter; SEM, standard error of means; ND, no detected.

1) C, no additive control; L, *Lactobacillus plantarum*; M, molasses; ML, molasses+*Lactobacillus plantarum*.

2) D, ensiling days; T, treatments; D×T, interaction between treatments and ensiling days.

* p<0.05.

a–d Values with different small letters show significant differences among ensiling days in the same treatment (p<0.05).

A–D Values with different capital letters show significant differences among treatments in the same ensiling days (p<0.05).

**Table 3:** Effect of additives and ensiling days on pH, dry matter, dry matter loss and ammonia nitrogen content of rice straw silages

Items	Treatment1)	Ensiling days				Means	SEM	Significance2)		
	
		6	15	30	60			D	T	D×T
pH	C	5.23^a^^A^	4.72^a^^A^	4.68^a^^A^	4.97^a^^A^	4.90^a^	0.06	*	*	NS
	L	4.14^b^^A^	3.81^b^^C^	3.91^bc^^B^	4.15^b^^A^	4.00^c^				
	M	4.30^b^^A^	4.03^b^^B^	4.23^b^^AB^	4.39^b^^A^	4.24^b^				
	ML	4.05^b^^A^	3.79^b^^B^	3.79^c^^B^	3.87^c^^B^	3.88^c^				
DM (g/kg FW)	C	385.39^b^^A^	374.51^c^^AB^	368.53^c^^BC^	354.20^b^^C^	370.66^b^	2.14	*	*	*
	L	395.39^ab^^A^	393.03^b^^A^	388.81^ab^^A^	385.50^a^^A^	390.68^a^				
	M	398.85^a^^A^	389.40^b^^B^	374.41^bc^^C^	361.08^b^^D^	380.94^a^				
	ML	404.25^a^^A^	400.10^a^^A^	395.64^a^^A^	393.61^a^^A^	398.40^b^				
DM loss (g/kg DM)	C	90.47^a^^C^	116.31^a^^BC^	139.58^a^^AB^	165.89^a^^A^	128.06^a^	5.48	*	*	*
	L	58.25^b^^B^	65.38^b^^B^	76.73^c^^AB^	91.66^b^^A^	73.00^c^				
	M	52.20^b^^D^	75.07^b^^C^	110.91^b^^B^	144.25^a^^A^	95.86^b^				
	ML	38.35^b^^A^	49.18^c^^A^	60.46^c^^A^	65.66^b^^A^	53.41^c^				
NH_3_-N (g/kg TN)	C	129.99^a^^C^	147.70^a^^BC^	164.23^a^^AB^	180.53^a^^A^	155.61^a^	5.11	*	*	NS
	L	61.99^d^^C^	72.56^c^^BC^	78.98^c^^AB^	92.46^c^^A^	76.50^c^				
	M	110.02^b^^C^	122.35^b^^BC^	134.13^b^^AB^	150.13^b^^A^	129.16^b^				
	ML	78.62^c^^B^	84.14^bc^^AB^	89.74^c^^AB^	99.44^c^^A^	87.99^c^				

SEM, standard error of means; DM, dry matter; FW, fresh weight; NH_3_-N, ammonia nitrogen; TN, total nitrogen.

1) C, no additive control; L, *Lactobacillus plantarum*; M, molasses; ML, molasses+*Lactobacillus plantarum*.

2) D, ensiling days; T, treatments; D×T, interaction between treatments and ensiling days.

* p<0.05; NS, not significant.

a–d Values with different small letters show significant differences among ensiling days in the same treatment (p<0.05).

A–D Values with different capital letters show significant differences among treatments in the same ensiling days (p<0.05).

**Table 4:** Effect of additives and ensiling days on structural carbohydrates composition of rice straw silages (g/kg DM)

Items	Treatment1)	Ensiling days				Means	SEM	Significance2)		
	
		6	15	30	60			D	T	D×T
NDF	C	659.02^a^^B^	662.80^a^^AB^	666.78^a^^AB^	670.32^a^^A^	664.73^a^	5.43	*	*	*
	L	620.71^b^^A^	612.95^b^^AB^	604.18^b^^B^	591.97^c^^C^	607.45^c^				
	M	626.10^b^	620.73^b^	608.98^b^	608.37^b^	616.05^b^				
	ML	615.80^b^^A^	605.83^b^^AB^	597.78^b^^AB^	582.36^c^^B^	600.44^c^				
ADF	C	413.75^a^^D^	417.37^a^^C^	423.32^a^^B^	427.70^a^^A^	420.54^a^	3.64	NS	*	*
	L	388.84^b^^A^	384.23^b^^AB^	380.54^b^^AB^	376.07^bc^^B^	382.42^bc^				
	M	392.59^b^	390.15^b^	381.04^b^	388.24^b^	388.01^b^				
	ML	385.59^b^^A^	383.09^b^^AB^	379.82^b^^BC^	371.84^c^^C^	380.09^c^				
ADL	C	63.55	64.46	66.90	69.20	66.03^a^	1.46	NS	*	NS
	L	60.63	61.53	61.69	62.65	61.62^ab^				
	M	60.32	62.24	64.91	67.49	63.74^ab^				
	ML	58.60	59.47	60.50	61.38	59.99^b^				
Cellulose	C	350.20^a^	352.92^a^	356.42^a^	358.50^a^	354.51^a^	3.41	*	*	NS
	L	328.21^b^^A^	322.70^b^^AB^	318.84^b^^AB^	313.42^b^^B^	320.80^b^				
	M	332.26^ab^	327.91^b^	316.13^b^	320.76^b^	324.26^b^				
	ML	327.00^b^^A^	323.62^b^^AB^	319.32^b^^AB^	310.46^b^^B^	320.10^b^				
Hemicellulose	C	245.27^a^	245.43^a^	243.46^a^	242.62^a^	244.19^a^	2.58	*	*	NS
	L	231.88^a^^A^	228.72^ab^^AB^	223.64^b^^B^	215.90^bc^^C^	225.03^bc^				
	M	233.52^a^	230.58^ab^	227.94^ab^	220.12^b^	228.04^b^				
	ML	230.20^a^^A^	222.74^b^^AB^	217.96^b^^AB^	210.52^c^^B^	220.36^c^				

DM, dry matter; SEM, standard error of means; NDF, neutral detergent fibre; ADF, acid detergent fibre; ADL, acid detergent lignin.

1) C, no additive control; L, *Lactobacillus plantarum*; M, molasses; ML, molasses+*Lactobacillus plantarum*.

2) D, ensiling days; T, treatments; D×T, interaction between treatments and ensiling days.

* p<0.05; NS, not significant.

a–c Values with different small letters show significant differences among ensiling days in the same treatment (p<0.05).

A–D Values with different capital letters show significant differences among treatments in the same ensiling days (p<0.05).

**Table 5:** Crude protein, ash and *in vitro* degradability of rice straw silage after 60 days of ensiling

Items	Treatment1)				SEM	Significance2)

	C	L	M	ML		
Crude protein (g/kg DM)	60.04^D^	62.24^C^	69.35^B^	72.79^A^	1.57	*
Ash (g/kg DM)	128.68^A^	121.82^B^	128.99^A^	126.46^A^	0.94	*
*In vitro* dry matter degradability (%)	56.36^B^	58.05^A^	56.27^B^	58.12^A^	1.57	*
*In vitro* neutral detergent fibre degradability (%)	48.21^B^	48.89^B^	50.63^AB^	52.33^A^	2.77	*
*In vitro* acid detergent fibre degradability (%)	47.37^A^	47.11^A^	47.88^A^	48.25^A^	3.54	NS

SEM, standard error of means; DM, dry matter.

1) C, no additive control; L, *Lactobacillus plantarum*; M, molasses; ML, molasses+*Lactobacillus plantarum*.

2) * p<0.05; NS, not significant.

A–D Values with different capital letters show significant differences among treatments (p<0.05).

**Table 6:** Effects of additives on *in vitro* fermentation characteristics of rice straw silages after 72 h incubation

Items	Treatment1)				SEM	Significance2)

	C	L	M	ML		
pH	6.81^A^	6.64^B^	6.70^AB^	6.61^B^	0.17	*
Acetic acid (mM)	40.04^C^	43.24^B^	42.54^B^	46.37^A^	1.57	*
Propionic acid (mM)	13.56^B^	13.51^B^	15.93^A^	16.64^A^	2.77	*
Isobutyric acid (mM)	0.53	0.62	0.76	0.84	0.14	NS
Butyric acid (mM)	5.99^A^	4.03^B^	3.16^C^	3.83^B^	1.51	*
Isovaleric acid (mM)	0.94	1.02	1.22	1.31	0.49	NS
Valeric acid (mM)	0.85	0.92	0.99	1.19	0.27	NS
Acetic acid/propionic acid	2.95^B^	3.20^A^	2.67^C^	2.79^BC^	0.33	*

SEM, standard error of means.

1) C, no additive control; L, *Lactobacillus plantarum*; M, molasses; ML, molasses+*Lactobacillus plantarum*.

2) * p<0.05; NS, not significant.

A–C Values with different capital letters show significant differences among treatments (p<0.05).
